# Editorial: Biomimetic control architectures for robots

**DOI:** 10.3389/fnbot.2022.998045

**Published:** 2022-08-16

**Authors:** Silvia Tolu, Egidio Falotico, Danish Shaikh, Eduardo Ros

**Affiliations:** ^1^Department of Electrical and Photonics Engineering, Technical University of Denmark, Kongens Lyngby, Denmark; ^2^The BioRobotics Institute, Scuola Superiore Sant'Anna, Pontedera, Italy; ^3^Department of Excellence in Robotics and AI, Scuola Superiore Sant'Anna, Pisa, Italy; ^4^University of Southern Denmark, Odense, Denmark; ^5^Department of Computer Architecture and Technology, University of Granada, Granada, Spain

**Keywords:** biomimetic control, neural networks, adaptive learning, modeling and control, bio-inspired control

Biological control systems are a source of inspiration for translating motor control principles into effective new designs for robot devices and controllers. Living organisms can perform many challenging tasks, e.g., simultaneous execution and tight coordination of multiple movements, the exploration of the surrounding physical environment, and adaptation to changing contexts. Nowadays, there is a wide variety of robots, ranging from rigid industrial robot arms to soft animal-like robots. The robotics community is working toward the ambitious goal of deploying these robots in unstructured and unpredictable, real-life scenarios. Brain-inspired control principles can be seen as effective solutions for these problems. Technologies based on these principles can enable robots to operate in such challenging scenarios and open the door to new applications beyond conventional control approaches. However, their potential is still unexplored in robotics.

This Research Topic was initiated to collect recent works on the development and experimental validation of biomimetic control architectures that combine biologically based approaches for the next generation of robotic systems. Overall, it comprises seven articles, five of which cover applications from the study to the design and modeling of bioinspired computing methods to achieve intelligence, flexibility, and adaptation for robotic applications such as locomotion, and manipulation. The remaining two articles regard the NeuroVis tool (Srisuchinnawong et al.) for real-time neural spatial-temporal information measurement and visualization, and a review on the whole-body nature of biological motor control that can inspire the design and control of future assistive systems (Seyfarth et al.).

A significant portion of the works is related to applications in locomotion. A locomotion controller based on central pattern generator (CPG) that integrates two adaptation mechanisms is presented in Thor et al.. The proposed control method enables online motor pattern adaptation during a locomotion task of a hexapod robot in a complex environment. The combination of frequency and motor pattern mechanisms can be seen as an essential step toward further studies on adaptive locomotion control. In Schmidt et al. the authors quantified the contributions that reflexes, and CPGs have on highly dynamic compliant movements and assessed the biomimetic robotic legs stability and energy efficiency under different environmental influences. This benchmark framework can help to improve future control strategies for robotics as well as generate testable hypotheses for implemented control mechanisms in biology.

Robots are also utilized for better understanding functional and computational models of different brain regions, and how the facilitate behaviors of living systems. Antonietti et al. presented a spiking computational model of the peripheral whisker system that was embedded in a virtual mouse neurorobot controlled by a cerebellar SNN. Future improvement of the model is expected to offer more advanced features, such as the recognition of surface textures, identification of movements of the touched object, or other complex touch-guided behaviors. Zahra et al. proposed a novel control system that integrates a motor cortex-like differential map transforming motor plans from task-space to joint-space and a static map correlating the joint spaces of a robot and a human teacher. The differential map is developed based on spiking neural networks while the static map is built as a self-organizing map. The integrated system allows a robot to mirror the actions performed by a human to its own joint space.

Learning-based techniques hold a strong potential for addressing the major challenges in robotics and they need to be adapted to different problems and applications. In order to guarantee fast online learning of the control parameters without the knowledge of a dynamic model of a real UAV system, a neural control method based on three-neuron network is presented in Jaiton et al.. In the future, neuromorphic implementations will allow the possibility of embedding even more realistic neural systems in physical robots. Finally, tools like NeuroVis (Srisuchinnawong et al.) will facilitate understanding embodied dynamics of neural information processes, boosts efficient neural technology development.

## Author contributions

All authors listed have made a substantial, direct, and intellectual contribution to the work and approved it for publication.

## Conflict of interest

The authors declare that the research was conducted in the absence of any commercial or financial relationships that could be construed as a potential conflict of interest.

## Publisher's note

All claims expressed in this article are solely those of the authors and do not necessarily represent those of their affiliated organizations, or those of the publisher, the editors and the reviewers. Any product that may be evaluated in this article, or claim that may be made by its manufacturer, is not guaranteed or endorsed by the publisher.

